# Thermally Optimized Polarization-Maintaining Photonic Crystal Fiber and Its FOG Application

**DOI:** 10.3390/s18020567

**Published:** 2018-02-13

**Authors:** Chunxi Zhang, Zhihao Zhang, Xiaobin Xu, Wei Cai

**Affiliations:** School of Instrument Science and Opto-electronics Engineering, Beihang University, Beijing 100191, China; zhangchunxi@buaa.edu.cn (C.Z.); xuxiaobin@buaa.edu.cn (X.X.); by1317142@buaa.edu.cn (W.C.)

**Keywords:** solid-core photonic crystal fiber, thermal performance, gyroscope

## Abstract

In this paper, we propose a small-diameter polarization-maintaining solid-core photonic crystal fiber. The coating diameter, cladding diameter and other key parameters relating to the thermal properties were studied. Based on the optimized parameters, a fiber with a Shupe constant 15% lower than commercial photonic crystal fibers (PCFs) was fabricated, and the transmission loss was lower than 2 dB/km. The superior thermal stability of our fiber design was proven through both simulation and measurement. Using the small-diameter fiber, a split high precision fiber optic gyro (FOG) prototype was fabricated. The bias stability of the FOG was 0.0023 °/h, the random walk was 0.0003 °/$\sqrt{h}$, and the scale factor error was less than 1 ppm. Throughout a temperature variation ranging from −40 to 60 °C, the bias stability was less than 0.02 °/h without temperature compensation which is notably better than FOG with panda fiber. As a result, the PCF FOG is a promising choice for high precision FOG applications.

## 1. Introduction

Photonic crystal fibers (PCFs) are fibers with a periodic transverse microstructure [1]. Since 1996, many kinds of PCFs have been designed and fabricated. The solid-core photonic crystal fiber is one type of photonic crystal fiber that guides light using total internal reflection [2]. The core region of a solid-core PCF is made of silica, and the core is surrounded by air hole cladding. Compared to conventional optical fiber, solid-core PCF has many unique properties. Research shows that when the air-filling fraction is below 0.43, the fiber only supports the fundamental mode: it the endless single mode [3]. As the solid-core PCF has less spurious twisting than ordinary polarization-maintaining (PM) fibers, the magnetic dependence can be reduced [4]. PM solid-core PCF (PM-PCF) utilizes shape birefringence. Hence, the birefringence of PCF can be adaptably designed according to various requirements [5,6]. The dispersion property of a PCF can be tailored through special design [7]. Furthermore, PCFs can be designed to have a small core to increase the nonlinear effects [8]. These characteristics make solid-core PCF an appropriate choice for fiber optic gyros (FOGs). Solid-core PCF gyros with an LD product equal to 2.9 in-km have been reported, and the measured bias error was less than 0.02 °/h [9]. Meanwhile, research on air-core PCF gyroscopes has demonstrated the substantial improvement these devices have on thermal stability, Kerr-induced drift and Faraday-effect-induced error [10]. However, a high scattering loss and its fabrication complexity makes this type of device unpractical in FOG applications. 

Apart from the aforementioned advantages, the thermal stability of the PCF is the key challenge. In FOG applications, phase sensitivity to temperature, known as the Shupe effect, causes the zero bias to drift, and the Shupe constant is utilized to characterize the phase variation [4]. According to a previous study by Song et al. [11], commercial PM-PCFs such as the PM-PCF of Yangtze Optical Fiber and Cable Joint Stock Limited Company (YOFC) and NKT Photonics [12,13] have a larger Shupe constant than traditional polarization-maintaining fibers, which may induce large thermal error, especially in high precision FOGs with a long fiber coil.

In this paper, we modified the parameters of the PM-PCF and studied the influence of these parameters on the Shupe constant. We then derived an optimized structure which has a reduced Shupe constant. Based on this design, we fabricated a small-diameter PM-PCF, and the Shupe coefficient of both the optimized PM-PCF and other commercial PM-PCFs were measured and compared. Based on the thermally optimized PM-PCF, a high precision FOG prototype was fabricated. The bias stability, thermal stability and scale factor stability were also tested.

## 2. Optimized Design of PM-PCF

In this section, we optimized the optical and thermal properties of the PM-PCF. It is critical that the key parameters should be verified as the fiber diameter reduces. Based on the optical optimized fiber structure, the thermal properties were studied theoretically. A PM-PCF with optimized parameters was fabricated, and, finally, the key parameters were measured.

### 2.1. Optimizing Optical Properties

**Figure 1:**
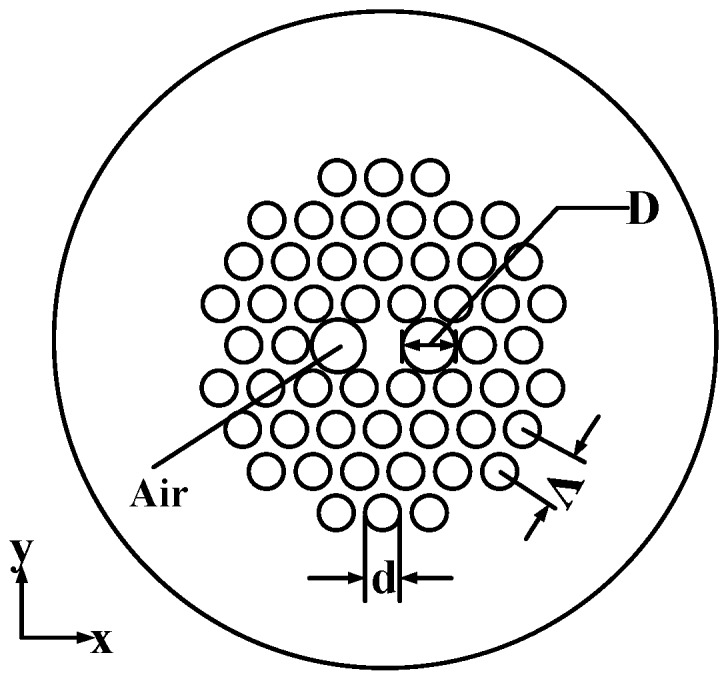
Parameters of a polarization-maintaining photonic crystal fiber (PCF) section.

In the context of FOG applications, we focused on the key parameters of mode-field diameter (MFD) matching, birefringence, and attenuation. The cross section of the solid core PM-PCF is shown in Figure 1, and the structure was adjusted to optimize the optical properties. In Figure 1, “d” is the diameter of the smaller air holes in the cladding and “D” is the diameter of the two enlarged air holes in the x-direction besides the fiber core. “Λ” is the center-to-center distance between two adjacent air holes.

We used the finite element method (FEM) to simulate how the key parameters change in response to varying the normalized frequency Λ/*λ*, air filling ratios d/Λ, and the diameter of the two enlarged air holes D [7]. The main characteristics of the optimization are shown in Table 1. The designed operational wavelength was 1550 nm, while the confinement loss at 1550 nm was lower than 0.01 dB/km. The MFD was modified to 6 μm by varying the d/Λ. This value of MFD is equal to the MFD of a panda fiber. The birefringence of the structure was 5 × 10^−4^.

### 2.2. Optimizing Thermal Properties

The Shupe constant (*S*) describes the phase variation due to temperature variation, which is the sum of the variation in fiber length and in the mode effective index per degree of temperature change. The constant is given as:

(1) $$S=\frac{1}{\phi }\cdot \frac{d\phi }{dT}=\frac{1}{n_{eff}}\cdot \frac{dn_{eff}}{dT}+\frac{1}{L}\cdot \frac{dL}{dT}=S_{n}+S_{L}$$

where *n_eff_* is the mode effective index and *L* is the fiber length, *S_n_* is the relative variation in the mode effective index per degree of temperature change and *S_L_* is the relative variation in fiber length per degree of temperature change. *S_n_* and *S_L_* are discussed separately below.

#### 2.2.1. Model of *Sn*

**Figure 2:**
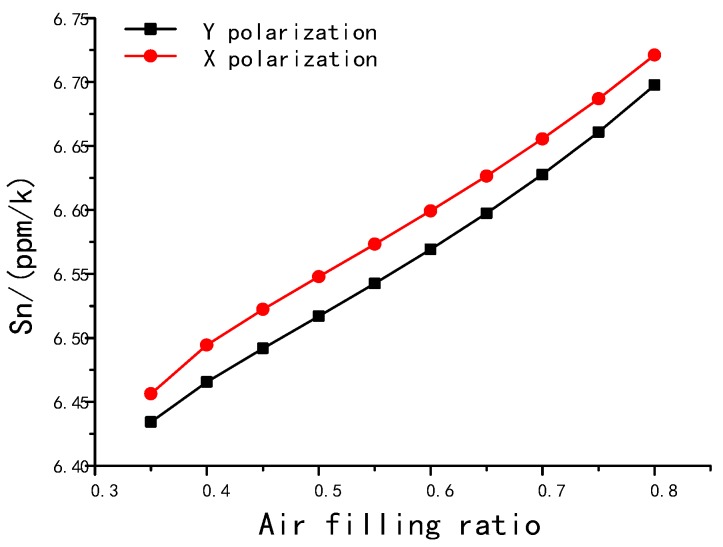
*S_n_* as functions of the air filling ratio.

In PM-PCFs, the mode effective index depends on the fiber section structure. We simulated the fiber section deformation due to the temperature change and the deformation-induced mode effective index change. The fiber structure was based on the aforementioned design. We built a 2D FEM model, taking into consideration the thermo-optic effect, the stress-optical effect, and section deformation. The *S_n_* was calculated in response to the changing section parameter. Key material parameters are listed in Table 2, while the fiber we studied has a single layer coating, and the coating material was polyimide. The simulation shown in Figure 2 indicates that *S_n_* increases with an increasing air-filling ratio. The *S_n_* of x polarization increases from 6.46 to 6.72 ppm/k as the air filling ratio changes from 0.35 to 0.8, while the *S_n_* of Y polarization increased from 6.43 to 6.70 ppm/k at the same time. Although changing the structure parameter contributed a 4% increase in *S_n_*, the thermo-optic coefficient, which is defined as the material’s refractive index change due to temperature, accounted for a large proportion of the change in *S_n_*.

#### 2.2.2. Model of *S_L_*

**Figure 3:**
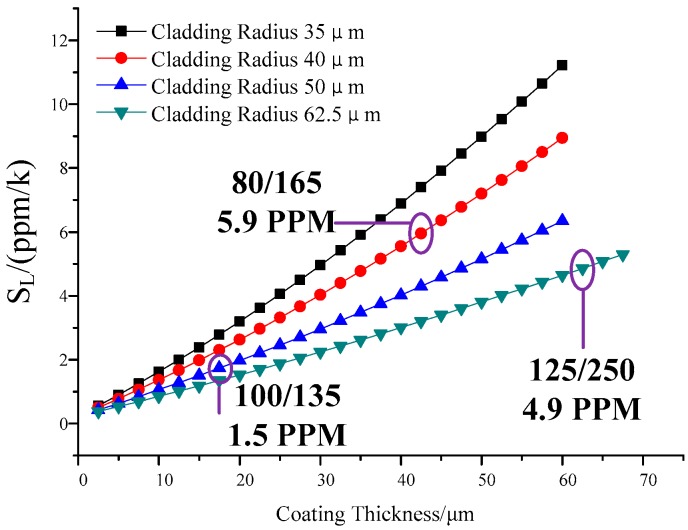
*S_L_* as a function of different fiber coating and cladding diameters.

The thermal expansion of the fiber core, cladding and coating together all result in the fiber length variation. Using the FEM method, we studied the *S_L_* of different fiber coating and cladding diameters. We built a 3D FEM model with the previously described parameters. We also determined the length change to calculate the *S_L_* with different fiber parameters. The result is shown in Figure 3. It can be seen that with a coefficient of thermal expansion α, *S_L_* grows larger as the coating diameter increases. With a constant coating thickness, *S_L_* decreases as the cladding diameter increases. As seen in Figure 3, fibers with standard cladding/coating diameters (80 μm/165 μm, 125 μm/250 μm, as marked on the figure) have a similar *S_L_* of around 5 ppm/k, and reducing the coating diameter is sufficient to reduce the *S_L_* of fibers with any cladding diameter. Taking into consideration the fiber strength, the *S_L_* could be decreased to 1 ppm/k by setting the cladding and coating diameters to 100 and 135 um, respectively.

**Figure 4:**
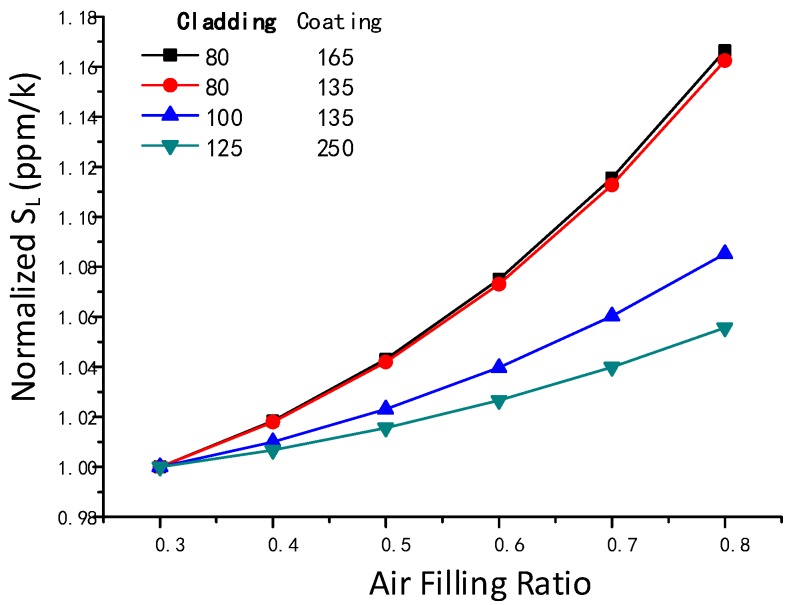
Normalized *S_L_* with different fiber diameters and air filling ratios.

We also calculated how the *S_L_* changes with different air filling ratios and different fiber diameters (Figure 4). Increasing the air filling ratio led to incremental increase in the *S_L_*, but the fiber with a larger cladding diameter exhibited a smaller increase in *S_L_*. Still, varying the air filling ratio had a small influence on the *S_L_*, as the *S_L_* changed from 0.3 to 0.8, with the maximum change in *S_L_* value being only 16%. In contrast, the *S* constant increased by 300% as the coating thickness varied from 5 to 60 um, as shown in Figure 3.

#### 2.2.3. Small-Diameter PM-PCF Structure with Optimized Thermal Properties

Considering all the factors, the section structure of the PM-PCF had little influence on both *S_n_* and *S_L_*. Decreasing the coating thickness while choosing a proper cladding diameter can sufficiently reduce the value of the *S* constant. Based on the optimized parameter derived above as well as the strength of the fiber, we fabricated a small-diameter PM-PCF. The cladding diameter is 100 μm and the coating diameter is 135 μm.

### 2.3. Fabricated Small-Diameter PM-PCF

**Figure 5:**
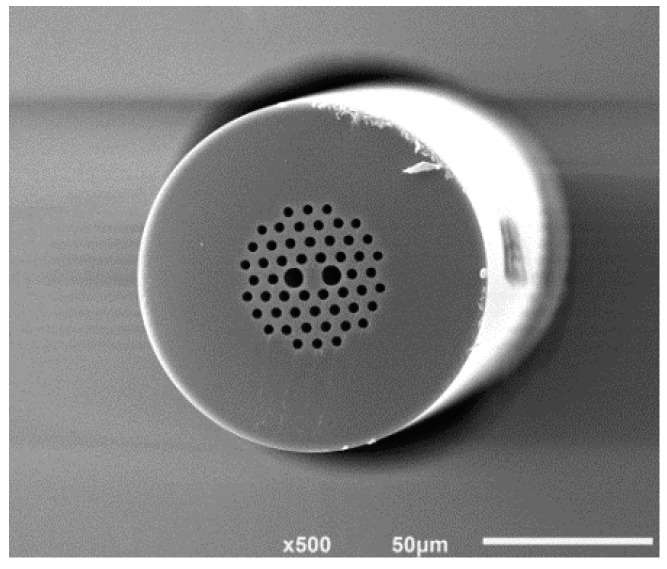
SEM picture of a small-diameter PM-PCF.

According to the previous analysis, a small-diameter PM-PCF with an optimized structure was fabricated in collaboration with the State Key Laboratory of Optical Communication Technologies and Networks Fiberhome. A scanning electron microscope (SEM) micrograph of the small-diameter PM-PCF section is shown in Figure 5.

**Figure 6:**
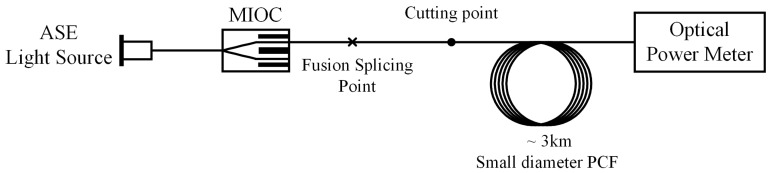
Cutback method of measuring transmission loss.

The small-diameter PM-PCF was up to 10 km in length and the transmission loss was as low as 2 dB/km at 1550 nm. The loss of fiber was measured using the cutback method, as shown in Figure 6. In this measurement, the ~3-km small-diameter PM-PCF is wound on a barrel ~16 cm in diameter. Broad band light from an ASE light source at 1550 nm is coupled to a multifunction integrated optic circuit (MIOC) chip with a distinction ratio of 50 dB. The pigtail of the MIOC is spliced to the fiber under test. Then, the polarized light passing through the fiber under test is collected by an optical power meter. The cutting point is after the splicing point and the splicing loss is eliminated. Furthermore, the fabricated fiber also showed a proof test level of 0.5%

### 2.4. Measurement of the S Constant

**Figure 7:**
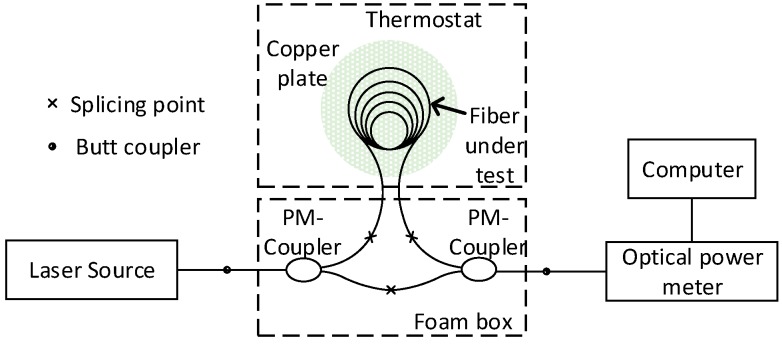
Mach–Zehnder interferometer to measure *S* constant.

A polarization-maintaining Mach–Zehnder interferometer was built to measure the parameter *S*, as shown in Figure 7. An NKT Photonics E15 polarization-maintaining laser source with an operating wavelength of 1550 nm and a line width of a few kilohertz was used. The laser output was butt coupled to a PM coupler. The fiber under test with a length of 4.4 m was circularly placed on a copper-plate to keep the fiber temperature uniform. The copper plate and fiber under test were placed in a thermostat. Each end of the fiber was spliced to a polarization-maintaining 3 dB coupler, and the two couplers were spliced to form the reference arm. A foam box was used to keep the reference part isolated from vibration and temperature fluctuation. An optical power meter was used to collect the light signal with a computer to record the power data.

**Figure 8:**
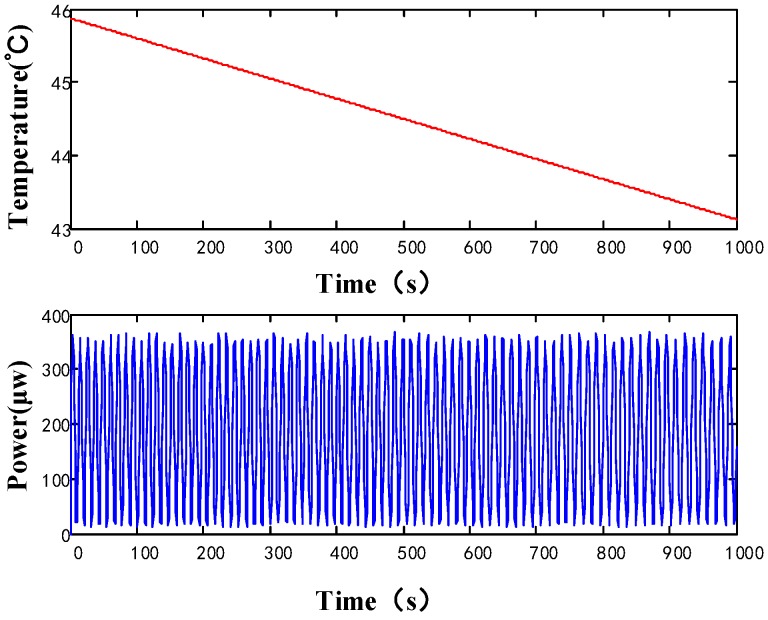
Optical power variation with temperature decrease.

To measure the *S* constant, the temperature of the thermostat was raised to 60 °C and kept steady until the system was stable. Then the thermostat was turned off, and left to cool down naturally and slowly to 20 °C. During this period, the optical path of the sensing arm changed as the refractive ratio of the tested fiber varied, and the power of the coherent light varied sinusoidally (Figure 8). The period of phase changing was recorded according to the period of the sinusoidal wave. Given the number of fringes *N*, the Shupe constant *S* can be calculated as:

(2) $$S=\frac{1}{\phi }\frac{d\phi }{dT}\approx \frac{1}{2\pi \frac{L}{\lambda }n_{eff}}\frac{\Delta \phi }{\Delta T}=\frac{N}{Ln_{eff}\Delta T}\cdot \lambda$$

where *λ* is the wavelength, *n_eff_* is the mode effective index, *L* is the length of fiber under test, and Δ*φ* is the phase-shift-difference between the sensing arm and reference arm. The *S* constants from different kinds of PCFs are shown in Table 3. 

The results demonstrate that the theoretical and experimental data agreed well, validating our finding that the small-diameter PM-PCF is more thermally stable than commercial PM-PCFs.

## 3. High Precision Solid-Core Photonic Crystal Fiber FOGs

### 3.1. High Precision FOG Setup

**Figure 9:**
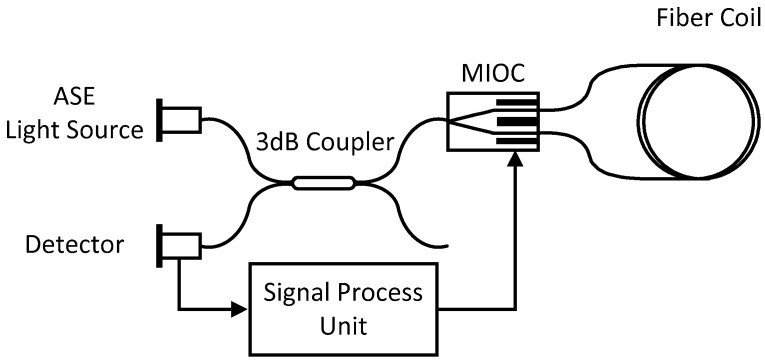
Schematic diagram of a small-diameter PM-PCF FOG.

**Figure 10:**
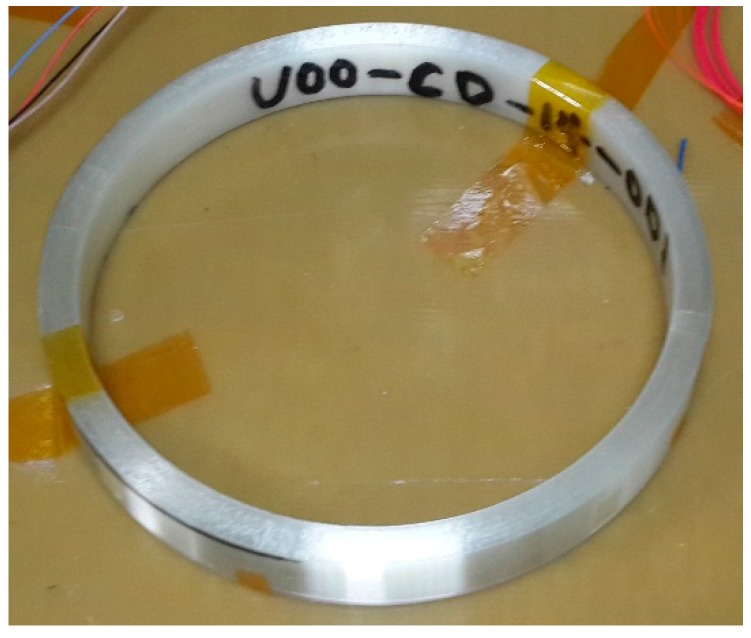
Picture of PCF coil wound by a small-diameter PCF.

We designed and fabricated a high precision FOG prototype with an optimized PM-PCF as mentioned above. The schematic diagram of the FOG is illustrated in Figure 9. A major difference between a PCF FOG and a traditional FOG is the sensing coil: a PCF FOG coil is wound by the PCF while a traditional FOG coil is wound by the panda fiber. Due to the air holes, the fiber strength was less than the panda fiber. A proof test was carried out with a force of 3.5 N. The proof test level of the PCF was 0.5%, while the proof test level of the panda fiber was 1%. The result showed that the PCF was more sensitive to force than the panda fiber, which means a more precise winding procedure is needed. Based on this test, we propose a high precision winding machine. A fiber coil of ~2000 m is quadrupole wound by the optimized PM-PCF, as shown in Figure 10.

Apart from the PCF fiber coil, other parts were also designed for the PCF FOG. An ASE light source with a spectral width of 15 nm and a central wavelength of 1550 nm was used. A high extinction ratio multifunction integrated optic circuit (MIOC) was used to maintain the polarization state and split the incident wave. The signal processing unit contained a 7π/8 and a four-state modulation scheme; both were used in the modulation process. 

For better thermal performance, a split FOG structure was used. The PCF sensing coil and the MIOC were placed in one structure referred to as the sensing coil system. The ASE light source, 3 dB coupler, detector, and signal processing unit were placed in another electric box. The box and sensing ring system were connected by a fiber and cable. As the light source and signal processing unit radiates heat, keeping them away from the sensing coil system can improve FOG performance.

### 3.2. FOG Performance Test

In order to test the high precision PCF FOG performance, we carried out a test at room temperature, and a full temperature test from −40 °C to 60 °C. FOG performance in response to different magnetic and vibration conditions was also tested.

#### 3.2.1. Room Temperature Test

**Figure 11:**
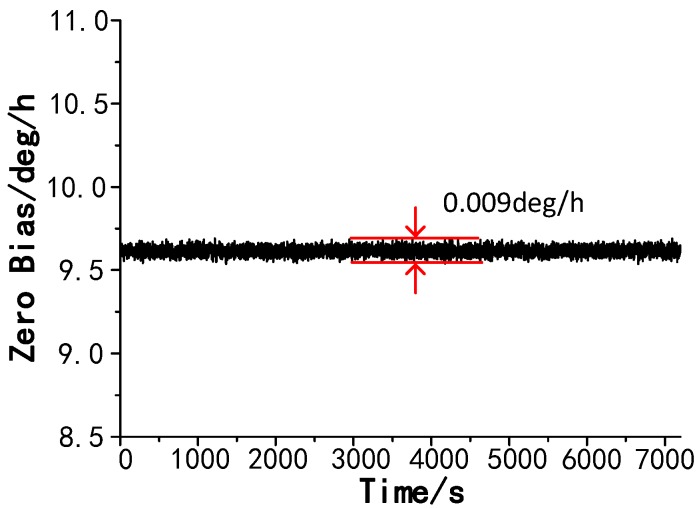
Output signal of the PM-PCF FOG recorded over a period of 7200 s at room temperature (integrated time is 1 s).

**Figure 12:**
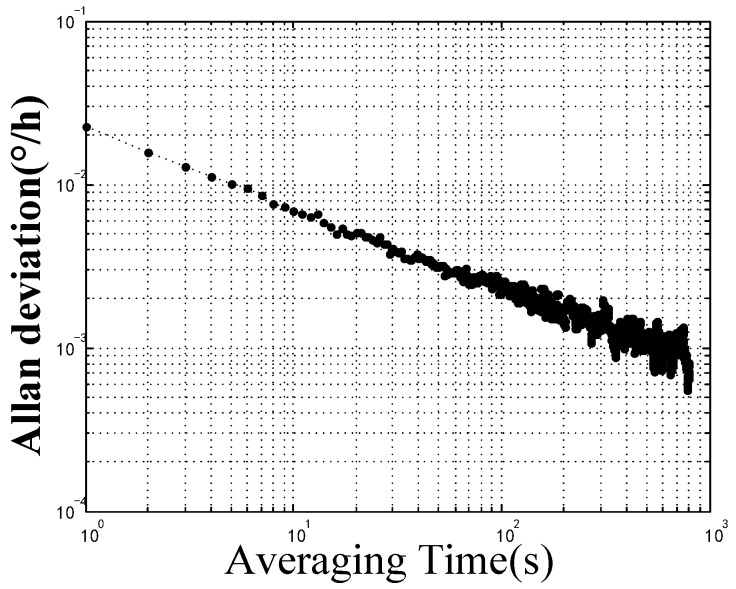
Allan deviation of the output signal of the PM-PCF FOG at room temperature.

The PCF FOG was placed on a marble platform with a vibration isolation foundation. The bias stability and bias repeatability at room temperature were tested. The output signal is demonstrated in Figure 11. The peak-to-peak value of the output signal approximates 0.009 °/h. The bias stability of this high precision coil was 0.0023 °/h (integration time was 100 s). The calculated angular random walk (ARW) was 0.0003 °/$\sqrt{h}$, as shown in Figure 12.

The scale factor was also tested at room temperature. The PCF FOG was placed on a turntable. The rotation speed was set at 0 °/s, ±0.5 °/s, ±1 °/s, ±2 °/s, ±5 °/s, ±10 °/s, ±20 °/s, ±50 °/s, ±100 °/s, 0 °/s, the duration of each test was 30 s. The FOG output was calculated as the average output of the FOG at each input angular velocity. If the zero bias value is removed from the output, the scale factor can be calculated by dividing the corresponding rotate speed. Based on the scale factor at each rotation speed, a derived nonlinearity scale factor was calculated of ~0.3 ppm and the scale factor asymmetry was ~0.3 ppm. After repeating the test seven times, the scale factor repeatability was tested and calculated as ~0.4 ppm.

#### 3.2.2. Full Temperature Test

**Figure 13:**
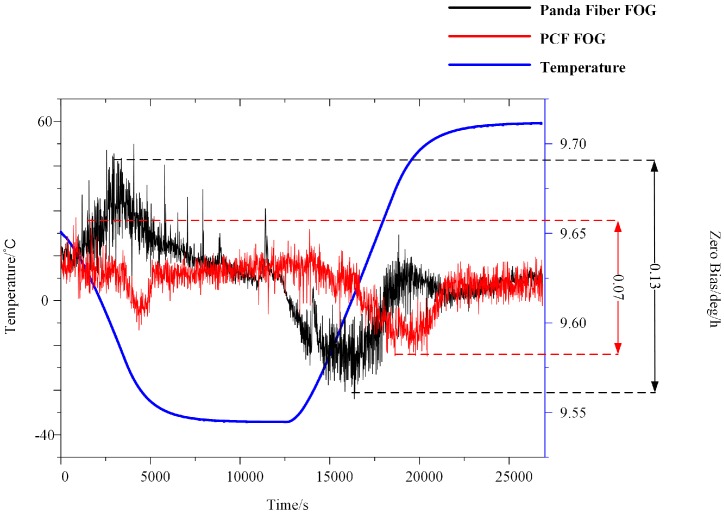
Output signal of two FOGs with different types of fiber with temperature varying from −40 to 60 °C.

The FOG was placed in a thermostat. The temperature was reduced from 20 to −40 °C and then raised up to 60 °C at a speed of 1 °C/min. the resulting FOG output without temperature compensation is shown in Figure 13. To compare the thermal performance of the small-diameter PM-PCF and the conventional panda fiber, we wound a panda fiber coil with the same diameter of the PCF coil. The output signals are shown in Figure 13, where the black curve represents the FOG of the panda fiber, the red curve represents the FOG of a small-diameter PM-PCF, and the blue curve stands for temperature. The peak-to-peak zero bias of the PCF FOG was 0.07 °/h, which was twice as good as the panda fiber FOG. With an integration time of 100 s, the bias stability throughout the entire procedure was 0.014 °/h. Though the Shupe constant of a small-diameter PM-PCF was only 5% smaller than that of a conventional panda fiber, a thinner cladding thickness and a smaller diameter allowed the fiber ring to reach thermal equilibrium more quickly.

## 4. Conclusions

We firstly studied the optical and thermal properties of a small-diameter PM-PCF. Key parameters were optimized to minimize the Shupe constant while keeping other optical properties in fulfilment of the FOG application requirements. According to simulations, we obtain an optimized small-diameter PM-PCF structure. Based on our optimization, we fabricated fiber with a cladding diameter of 100 μm and a coating diameter of 135 μm. The Shupe constant of this small-diameter PM-PCF was tested using a Mach–Zehnder interferometer and the results were compared with an NKT PM-1550 PCF. The *S* constant of our small-diameter PM-PCF was 15% lower than that of commercial PCFs. 

Based on the fabricated PCF, a split high precision FOG prototype was fabricated. The bias stability of the FOG was 0.0023 °/h, the random walk was 0.0003 °/$\sqrt{h}$, and the scale factor error was less than 1 ppm. Throughout temperatures varying from −40 to 60 °C, the bias stability was less than 0.02 °/h without temperature compensation, which is notably better than FOGs with panda fibers. As a result, the PCF FOG is a promising choice in high precision FOG applications.

## Figures and Tables

**Table 1:** Main characteristics of the PCF section.

Parameter	Value
d/Λ	0.56
Λ/*λ*	3.60
D	5.70

**Table 2:** Key parameters of materials [14].

	Thermal Expansion Coefficient (ppm)	Poisson’s Ratio (1)	Young’s Modulus (GPa)	Thermal-Optical Coefficient (1/k)
Cladding (Silica)	0.56	0.17	72.4	6 × 10^−6^
Coating (Acrylate)	80	0.4	0.8	—

**Table 3:** Measured and calculated Shupe constant of tested fiber.

	Shupe Constant	
	Calculation	Measured
NKT PM-1550	11.46	8.18 ± 0.1
Small-diameter PM-PCF	8.34	7.45 ± 0.1
